# Experimental and regional variations in Na^+^‐dependent and Na^+^‐independent phosphate transport along the rat small intestine and colon

**DOI:** 10.14814/phy2.12281

**Published:** 2015-01-27

**Authors:** Joanne Marks, Grace J. Lee, Sobiya P. Nadaraja, Edward S. Debnam, Robert J. Unwin

**Affiliations:** Department of Neuroscience, Physiology & Pharmacology, University College London, London, UK; UCL Centre for Nephrology, University College London, London, UK

**Keywords:** Luminal phosphate concentration, NaPi‐IIb, regional phosphate absorption

## Abstract

Despite the importance of extracellular phosphate in many essential biological processes, the mechanisms of phosphate transport across the epithelium of different intestinal segments remain unclear. We have used an in vitro method to investigate phosphate transport at the brush border membrane (BBM) of intact intestinal segments and an in vivo method to study transepithelial phosphate absorption. We have used micromolar phosphate concentrations known to favor NaPi‐IIb‐mediated transport, and millimolar concentrations that are representative of the levels we have measured in luminal contents, to compare the extent of Na^+^‐dependent and Na^+^‐independent phosphate transport along the rat duodenum, jejunum, ileum, and proximal and distal colon. Our findings confirm that overall the jejunum is the main site of phosphate absorption; however, at millimolar concentrations, absorption shows ~30% Na^+^‐dependency, suggesting that transport is unlikely to be mediated exclusively by the Na^+^‐dependent NaPi‐IIb co‐transporter. In the ileum, studies in vitro confirmed that relatively low levels of phosphate transport occur at the BBM of this segment, although significant Na^+^‐dependent transport was detected using millimolar levels of phosphate in vivo. Since NaPi‐IIb protein is not detectable at the rat ileal BBM, our data suggest the presence of an as yet unidentified Na^+^‐dependent uptake pathway in this intestinal segment in vivo. In addition, we have confirmed that the colon has a significant capacity for phosphate absorption. Overall, this study highlights the complexities of intestinal phosphate absorption that can be revealed using different phosphate concentrations and experimental techniques.

## Abstract (precis)

We have used in vitro and in vivo methods to investigate phosphate absorption in different regions of the rat small and large intestine at micromolar and millimolar phosphate concentrations. Our findings confirm that overall the jejunum is the main site of phosphate absorption but at millimolar concentrations phosphate absorption also occurs in the ileum and colon. Overall, this study highlights the complexities of intestinal phosphate absorption that can be revealed using different phosphate concentrations and experimental techniques.

## Introduction

Phosphate homeostasis is maintained by mechanisms that mediate and control phosphate transport across the renal and intestinal epithelium. In the steady state the kidneys excrete phosphate at the same rate as that absorbed by the small intestine. However, despite our detailed knowledge of the mechanisms and regulation of renal phosphate transport in the kidney, we know far less about the pathways responsible for phosphate absorption across the intestinal tract. Moreover, conflicting views on intestinal phosphate transport are likely to reflect species differences concerning the primary location of phosphate absorption along the intestinal tract (Marks et al. 2006) and the variety of experimental techniques used to assess phosphate uptake, as well as the widely differing phosphate concentrations added to uptake buffers. Indeed, information as fundamental as the postprandial luminal phosphate level is not readily available.

Early studies of phosphate uptake using intestinal BBM vesicles revealed a process that was critically dependent on sodium, with an affinity for phosphate of ~0.1 mmol/L (Berner et al. 1976; Loghman‐Adham et al. 1987). The murine type II phosphate transporter, NaPi‐IIb, was later cloned and characterized; both isotope flux and electrophysiological measurements in Xenopus oocytes revealed an apparent *K*_m_ for phosphate of ~50 *μ*mol/L and a *K*_m_ for sodium of ~30 mmol/L (Hilfiker et al. 1998). This finding led to the proposal that NaPi‐IIb is responsible for active and saturable phosphate transport at the intestinal BBM.

It is now clear that the profile of phosphate absorption along the rat and mouse small intestine displays some important differences (Radanovic et al. 2005; Marks et al. 2006). Studies using the in situ intestinal loop technique, with phosphate concentrations close to the apparent *K*_m_ of NaPi‐IIb for phosphate, have shown that maximal absorption across the rat small intestine is seen in the duodenum and jejunum, with very little phosphate absorption occurring in the ileum (Marks et al. 2006); this is similar to the profile in human small intestine (Walton and Gray 1979; Borowitz and Ghishan 1989). In contrast, phosphate is absorbed along the entire mouse small intestine in vivo, with the highest rate occurring in the ileum (Marks et al. 2006). In vitro studies using everted sacs (Sabbagh et al. 2009) and BBM vesicles (Radanovic et al. 2005; Giral et al. 2009) have confirmed these species differences, and it is now recognized that the profile of active phosphate absorption correlates with the levels of NaPi‐IIb mRNA and protein, with highest levels seen in the rat proximal and mouse distal small intestine (Radanovic et al. 2005; Marks et al. 2006; Giral et al. 2009).

In this context, recent studies using conditional tamoxifen‐inducible NaPi‐IIb^−/−^ knockout mice have reported that NaPi‐IIb‐mediated transport in the mouse ileum accounts for ~90% of its total Na^+^‐dependent phosphate absorption across the BBM. However, this study also revealed that the transporter accounts for only ~50% of total intestinal transepithelial phosphate absorption in response to a dietary phosphate load (Sabbagh et al. 2009). In addition, inhibition of NaPi‐IIb in rats by oral administration of phosphonoformic acid (PFA) had no effect on Na^+^‐dependent BBM phosphate transport in animals maintained on a normal phosphate diet, although it did blunt the increased Na^+^‐dependent phosphate transport induced by a low phosphate diet (Loghman‐Adham et al. 1993). Taken together these studies raise the possibility of an additional non‐NaPi‐IIb‐mediated pathway for phosphate absorption.

The concept of an alternative pathway for phosphate absorption is not new: numerous studies in vivo using luminal phosphate concentrations of up to 100 mmol/L revealed a linear relationship between phosphate concentration and its absorption, with no evidence for a saturable process (Walton and Gray 1979; Davis et al. 1983; Aloia and Yeh 1985; Williams and DeLuca 2007). These and related studies concluded that paracellular transport was the dominant absorption pathway at high luminal phosphate concentrations in both rodents and humans.

While the debate continues over the exact pathways responsible for intestinal phosphate absorption, it is widely recognized that the small intestine is responsible for most phosphate absorption. However, interestingly, the original studies characterizing NaPi‐IIb demonstrated the presence of this gene transcript in mouse colon, as well as the small intestine (Hilfiker et al. 1998). In addition, significant paracellular phosphate transport has been reported in the rat distal colon (Hu et al. 1997). These observations, as well as the finding that in man phosphate‐containing enemas can induce hyperphosphatemia, suggests a pathway for the rapid translocation of phosphate across the colonic mucosa (Hunter et al. 1993; Hu et al. 1997; Carl and Mitchell 2007).

The aim of the present study was to address some of these apparent discrepancies and uncertainties arising from previous studies of intestinal phosphate absorption. We measured unbound phosphate levels in luminal contents collected from specific intestinal regions of rats maintained on a normal phosphate diet. Based on these values, in vitro and in vivo methods were then used to determine the contribution of Na^+^‐dependent and Na^+^‐independent phosphate absorption in defined regions of small intestine. The contribution of the colon to phosphate uptake and the pathways involved were also investigated.

## Materials and Methods

### Animals

Male Sprague–Dawley rats of 6–8 weeks of age were obtained from Charles River Laboratories (Harlow, UK) and used in accordance with the UK Animals (Scientific Procedures) Act, 1986, Amendment Regulations 2012. Rats were fed with normal rat chow containing 0.52% phosphate (Diet RM1; SDS Ltd, Witham, Essex, UK).

### Phosphate level in the intestinal lumen

Rats were anesthetized with an intraperitoneal (i.p.) injection of pentobarbitone sodium (45 mg/kg, Pentoject; Animalcare Ltd, York, UK) 3 h after the transition to light, in the 12 h light/12 h dark cycle employed in the biological services unit at the Royal Free Campus. Intestinal contents were squeezed out of defined regions of the intestinal tract; duodenum (beginning at stomach and ending at the ligament of Treitz), jejunum (beginning at the ligament of Treitz and ending half way along the remainder of the small intestine), ileum (the remaining section of small intestine ending at the cecum), proximal colon (the first half of the colon distal to the cecum), and distal colon (the second half of the colon) and diluted 1:1 with distilled water and vortexed vigorously. Solid contents from ileum and colon were diluted 1:1 and homogenized for 10 sec (Ultra Turrax homogeniser; Janke & Kunkel, Staufen, Germany). All samples were centrifuged at 6000 rpm for 10 min and an aliquot of the supernatant was used to determine free (unbound) phosphate concentration using a Quantichrom phosphate assay kit (BioAssay Systems, Hayward, CA, USA), according to the manufacturer's instructions.

### Phosphate uptake in vitro using the everted sleeve technique

Rats were anesthetized with an i.p injection of pentobarbitone sodium. Segments (2–4 cm long) of duodenum (beginning 1 cm from stomach), jejunum (beginning at the ligament of Treitz), ileum (5 cm proximal to the cecum), proximal colon (2 cm distal to the cecum), and distal colon (4 cm proximal to the anus) were flushed through with saline at room temperature and everted using a glass rod. The tissue was secured to the rod with thread and preincubated for 5 min at 37°C in NaCl‐HEPES buffer (pH 7.4) containing in mmol/L: 16 Na^+^‐HEPES, 3.5 KCl, 10 MgSO_4_, 1 CaCl_2_, and 125 NaCl, and gassed with 100% O_2_ and stirred continuously. The tissue was then incubated for 2 min in oxygenated and stirred preincubation buffer with the addition of 10 mmol/L glucose, 0.1–10 mmol/L KH_2_PO_4_ and 0.74 MBq ^32^P (Perkin Elmer, Bucks, UK). Na^+^‐free uptake buffer was made using Na^+^‐free HEPES, with choline chloride (ChCl) used as an iso‐osmotic substitute for NaCl. To prevent Ca/Pi precipitation when higher KH_2_PO_4_ concentrations were used, 1 mmol/L CaCl_2_ was added to all uptake buffers. Washing the tissue with 10‐fold excess of nonradioactive phosphate in 150 mmol/L NaCl, or ChCl, for 10 min, followed by PBS for 10 min terminated the uptake of ^32^P. These conditions were optimized to ensure uptake was measured during the linear phase of transport and that measurement of radioactive phosphate represents phosphate accumulation in the tissue, rather than that bound to the mucosa surface (Marks et al. 2007). Tissue (~100 mg) was weighed and digested in 2 mL of Solvable (Perkin Elmer). Aliquots (100 *μ*L) of the digested sample and initial uptake solution were counted in Ultima‐Gold XR scintillation fluid (Perkin Elmer) using a scintillation counter (Tri‐Carb 2900TR; Perkin Elmer) to permit calculation of phosphate retention of the different intestinal segments. Results were expressed as nmoles of phosphate per 100 mg of intestinal tissue.

### Phosphate absorption in vivo

The in situ ligated intestinal loop technique was used to quantify transepithelial phosphate absorption, as has been reported previously (Marks et al. 2006) in rats anesthetized as described above. In brief, 500 *μ*L of uptake buffer (containing in mmol/L: 16 Na^+^‐HEPES or Na^+^‐free HEPES, 140 NaCl or ChCl, 3.5 KCl, 0.1–10 KH_2_PO_4_, and 0.37 MBq ^32^P) was instilled into a 5 cm long segment of duodenum, jejunum, ileum, and proximal or distal colon, selected as described above. During this procedure animal body temperature was maintained at 37°C using a heating blanket. Blood was collected after 10, 20, and 30 min via a femoral artery cannulation to ensure uptake was linear and the ^32^P activity in the plasma counted. The rates of transepithelial transport (^32^P appearance in plasma) were calculated, taking into account the initial ^32^P activity in the luminal buffer and expressed as nmoles of phosphate transferred into 1 mL plasma by 1 g of intestine at 30 min.

### Sodium concentration in luminal fluid

Sodium‐free uptake solutions prepared by isosmotic replacement of Na^+^ with ChCl were instilled into cannulated sections of small intestine in vivo, as described above. After 30 min incubation, the Na^+^ concentration in the luminal solution was measured using CoroNa^™^ Green dye (Invitrogen, Paisley, UK) to assess the degree of Na^+^ secretion into initially Na^+^‐free uptake buffer. A calibration curve was obtained using known concentrations of Na^+^ up to 25 mmol/L and ChCl‐HEPES buffer was used as a blank; 0.25 *μ*mol/L of dye was added to 100 *μ*L of standard and sample solutions, and fluorescence was read at 492 nmol/L using a spectrofluorimeter (SFM 25; Kontron Instruments, Zurich, Switzerland).

### Electron Microscopy

Pieces of small or large intestine taken at the end of the in vitro or in vivo experiments (approximately 1 cm) were fixed for 24 h in an ice‐cold 1.5% glutaraldehyde/1% paraformaldehyde mixture (pH 7.2–7.4). Tissue was postfixed in a 1% osmium tetroxide/1.5% potassium ferrocyanide mixture and then dehydrated through a graded series of alcohols (70, 90, 100%) and further processed through Lemix resin (TAAB Labs, Berks, UK) using a 50/50 mixture with ethanol overnight followed by 100% resin for 24 h. The tissue pieces were embedded in polythene capsules and cured overnight at 65°C. Sections were initially cut at approximately 0.5–1 mm to find the area of interest, then cut into ultra‐thin sections (~70 nm) and mounted on copper grids. Sections were stained with 2% uranyl acetate and Reynold's lead citrate, and examined with a Japanese Electron Optics Laboratories electron microscope (model 1200EX; JEOL (UK) Ltd, Herts, UK). Tight junctions were examined at a final magnification of between 12–50,000×.

### Statistical analysis

Data are presented as means ± SEM. All statistical comparisons were made using unpaired *t*‐tests with statistical significance taken as *P *<**0.05; *n* is the number of observations per group using tissue taken from different animals.

## Results

### Luminal phosphate concentration

Information as fundamental as the phosphate level found in intestinal luminal contents is not readily available. This has led to most studies of intestinal phosphate handling employing phosphate concentrations of 0.1 mmol/L to reflect the affinity of intestinal BBM vesicles for phosphate documented in the early studies (Berner et al. 1976; Loghman‐Adham et al. 1987), and the known kinetic characteristics of NaPi‐IIb when expressed in oocytes (Hilfiker et al. 1998; Forster et al. 2006). However, some have used low millimolar (Kirchner et al. 2008; Douard et al. 2010) or even molar phosphate concentrations (Berndt et al. 2007; Williams and DeLuca 2007), making it difficult to compare the findings of these different studies. Therefore, we measured the free (unbound) phosphate concentration in luminal contents collected from specific regions of the intestinal tract in rats maintained on a normal (0.52%) phosphate diet. Interestingly, phosphate levels in all regions studied were in the millimolar range (Table 1); the mean value in small intestinal luminal fluid was 6.3 mmol/L ‐ higher levels were found in the stomach and distal colon.

**Table 1.** Unbound phosphate concentration in the luminal contents removed from specific regions of small and large intestine. Values are means ± SEM;* n *=**6–8

Intestinal segment	Free luminal phosphate concentration (mmol/L)
Stomach	14.84 ± 3.43
Duodenum	7.04 ± 1.50
Jejunum	5.69 ± 0.37
Ileum	6.27 ± 0.86
Distal Colon	11.49 ± 0.62

### Phosphate transport in the duodenum and jejunum in vivo and in vitro

**Figure 1.**
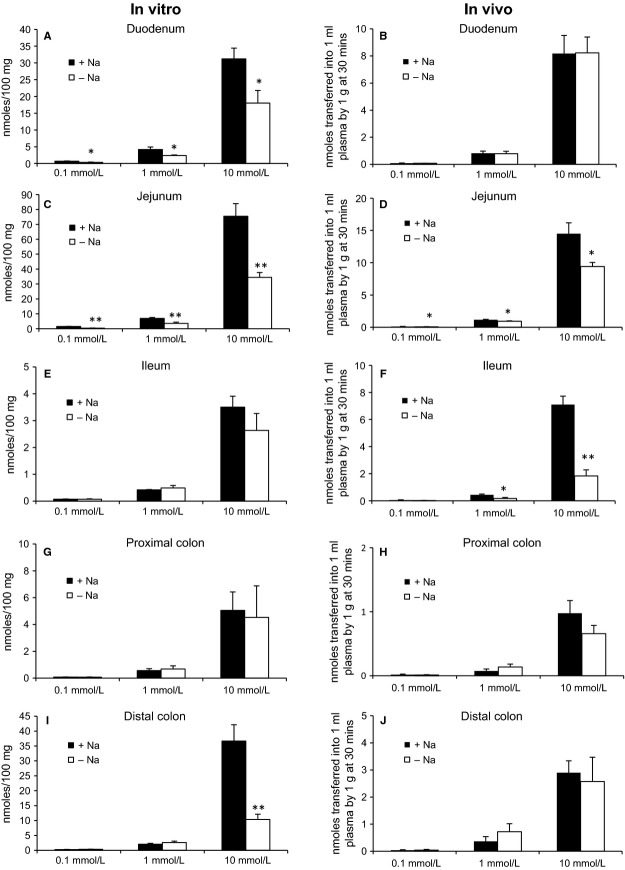
Phosphate transport measured in vitro *and* in vivo using duodenal, jejunal and ileal regions of small intestine, and proximal and distal colon. Total phosphate transport in the presence of Na^+^ (solid bars) and Na^+^‐independent transport in the presence of ChCl (open bars) was determined using buffers containing phosphate concentrations of 0.1, 1, and 10 mmol/L. Na^+^‐dependent uptake is representative of total transport minus that observed following replacement of NaCl with ChCl. Data are means ± SEM. *P < 0.05, **P < 0.01 compared with absorption in the same region in the presence of Na^+^ using unpaired *t*‐test; *n *=**6–9.

Phosphate transport in the duodenum and jejunum was measured both in vivo and in vitro using animals maintained on a normal phosphate diet. Experiments were initially carried out using uptake buffer containing 0.1 mmol/L phosphate with or without Na^+^. As previously reported in the rat, phosphate absorption occurred predominantly in the jejunum (Fig. 1C and D), with lower rates of uptake in the duodenum (Fig. 1A and B). In the jejunum, Na^+^‐dependent transport accounted for 73 ± 5% of total phosphate transport measured in vitro (Fig. 1C), but only 32 ± 8% of transport in vivo (Fig. 1D). In the duodenum, 48 ± 12% (Fig. 1A) of phosphate transport in vitro was Na^+^‐dependent, although surprisingly Na^+^‐dependent phosphate transport was not evident in this segment under in vivo conditions (Fig. 1B).

Based on the luminal phosphate concentrations observed in rats fed a normal maintenance diet, we examined the effect of millimolar phosphate levels in the uptake buffer on Na^+^‐dependent and Na^+^‐independent phosphate transport. In vitro*,* phosphate uptake rose with increasing phosphate concentrations and was mediated by Na^+^‐dependent and Na^+^‐independent mechanisms in the duodenum (Fig. 1A) and jejunum (Fig. 1C). In the duodenum, Na^+^‐dependent phosphate transport in vitro remained constant, regardless of the phosphate concentration present in the uptake buffer, while in the jejunum the contribution of Na^+^‐dependent transport declined from 73 ± 5% of total phosphate transport at 0.1 mmol/L to 53 ± 3% at 10 mmol/L phosphate.

Similar to the results noted in vitro, in the duodenum and jejunum total transepithelial phosphate absorption in vivo rose as the phosphate concentration in the uptake buffer was increased. Only a Na^+^‐independent pathway‐mediated phosphate absorption in the duodenum in vivo at all phosphate concentrations tested (Fig. 1B). In the jejunum, in contrast to measurements in vitro, Na^+^‐dependent phosphate transport in vivo remained relatively stable, accounting for 32 ± 8% of uptake at 0.1 mmol/L and 29 ± 10% at 10 mmol/L luminal phosphate (Fig. 1D).

### Does sodium secretion into the intestinal lumen, or changes in tight junction integrity, influence Na^+^‐independent transport?

The Na^+^ concentration of the uptake buffer at the end of the in vitro and in vivo uptake period was measured to determine whether the higher proportion of Na^+^‐independent transport seen in vivo was partially driven by Na^+^ secretion into the uptake buffer. Na^+^ was undetectable following incubation with initially Na^+^‐free buffer in vitro, suggesting that the higher proportion of Na^+^‐independent uptake seen at 10 mmol/L versus 0.1 mmol/L phosphate in the jejunum was not due to Na^+^ secretion during the 2 min incubation period. In contrast, Na^+^ was detected in buffer collected at the end of the 30 min in vivo uptake period (11.9 ± 2.8 mmol/L). Although this value is lower than the reported *K*_m_ of NaPi‐IIb for Na^+^ of ~30 mmol/L (Hilfiker et al. 1998), it may be sufficient to drive some Na^+^‐dependent phosphate transport under Na^+^‐free uptake conditions.

**Figure 2.**
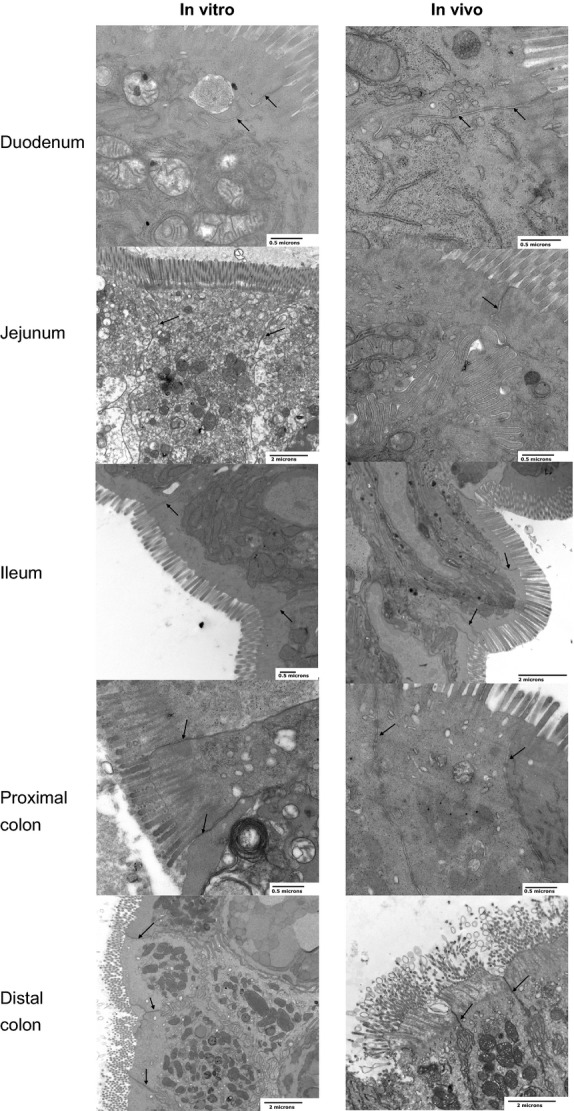
Electron Microscope images of different regions of the small and large intestine processed following in vitro or in vivo measurement of phosphate uptake. Tight junctions are indicated by arrows (scales bars = 0.5 or 2 microns).

Electron microscope images revealed that manipulation of segments of small or large intestine for phosphate uptake in vivo or in vitro had no effect on the integrity of the tissues (Fig. 2). These findings confirm that damage to the tissue, particularly in the region of the tight junctions cannot explain the differences in Na^+^‐independent transport measured using the two experimental approaches.

### Ileal phosphate transport in vivo and in vitro

Previous studies have shown very low values for phosphate transport and undetectable gene and protein expression for NaPi‐IIb in the rat ileum (Marks et al. 2006; Giral et al. 2009). The present study confirms that using 0.1 mmol/L phosphate, minimal ileal phosphate transport occurs in vitro (Fig. 1E) and in vivo (Fig. 1F) with or without Na^+^ in the uptake buffer. In vitro, transport increased with millimolar concentrations of phosphate, but this corresponded to only 12 ± 2 and 4.9 ± 0.6% of total transport in the duodenum and jejunum, respectively; Na^+^‐dependent transport was not detected. In contrast, the rate of transepithelial phosphate absorption in the ileum in vivo using millimolar phosphate concentrations was similar to that seen in the duodenum, and unexpectedly, Na^+^‐dependent phosphate transport was shown to account for 52 ± 22 and 74 ± 5% at 1 and 10 mmol/L phosphate, respectively (Fig. 1F).

### Phosphate transport in the colon in vivo and in vitro

Paradoxically, previous studies have demonstrated the presence of NaPi‐IIb gene transcript in mouse colon (Hilfiker et al. 1998), while significant levels of paracellular phosphate transport have been reported in the rat distal colon (Hu et al. 1997). The present study has shown that phosphate absorption in vitro and in vivo is consistently higher in the distal colon compared with the proximal colon. Transepithelial phosphate absorption in the distal colon in vivo was 30 ± 6% of that seen in the duodenum at 10 mmol/L, confirming that this segment has the capacity for phosphate absorption (Fig. 1J). Interestingly, in vitro*,* Na^+^‐dependent phosphate transport was detectable at 10 mmol/L phosphate, with total transport levels similar to those seen in the duodenum (Fig. 1I).

## Discussion

The mechanisms and regulation of intestinal phosphate absorption remain poorly defined. This is partly due to known species differences, as well as the variety of techniques and experimental conditions that have been used to assess phosphate absorption, particularly the wide range (and largely unphysiological choice) of phosphate concentrations added to uptake buffers. In the present study we have determined the luminal concentration of phosphate available for absorption, and we have used in vivo and in vitro methods to characterize the relative contributions of Na^+^‐dependent and Na^+^‐independent phosphate transport along the rat small and large intestine.

We have used the in vitro, everted sleeve technique developed by Karasov and Diamond (Karasov et al. 1983), and optimized by ourselves for the measurement of phosphate transport at the enterocyte BBM (Marks et al. 2007). Phosphate uptake was measured within the linear phase to ensure unidirectional flux, and that the isotope had not reached the serosal surface, while allowing time for equilibrium of the tissue with the buffer and ensuring that uptake was readily detectable. Our previous studies combining the everted sleeve technique with autoradiography confirmed that the integrity of the villi was unaffected by the process of eversion (Marks et al. 2007), and established that tissue handling was not likely to affect the interpretation of our results (Starck et al. 2000). The in situ ligated intestinal loop technique was used to quantify phosphate absorption in vivo. In contrast to the everted sleeve technique, measurement of phosphate transport in vivo provides an estimate of transepithelial phosphate absorption and is influenced by the local circulation, enteric nervous system, and transmural potential difference. We have previously validated this technique for the measurement of transepithelial phosphate absorption (Marks et al. 2006), and shown that limiting the volume of uptake solution instilled into the segment to 0.5 mL avoids the effect of intraluminal distention on intestinal function, and that collecting blood via a femoral artery cannulation after 10, 20, and 30 min ensures uptake is in the linear phase.

There is very little published information on the luminal “free” phosphate concentration available for absorption following ingestion of a normal diet. In weaning rats, the phosphate level in the upper small intestinal lumen has been reported to be ~5 mmol/L (Kirchner et al. 2008), and our own data show similar values for the luminal contents found in the small intestine of adult rats. In humans, phosphate levels in aspirated jejunal fluid have been documented to be between 0.5 and 17.5 mmol/L, depending on whether a low or high phosphate meal was ingested (Davis et al. 1983). While differences in phosphate content of the diet clearly affect luminal phosphate concentration, it is also recognized that the type of phosphate can significantly affect the overall rate of dietary phosphate absorption: inorganic phosphate used as a preservative in processed foods appears to have much higher bioavailability, resulting in >90% absorption, compared with only 40–60% for naturally occurring dietary phosphate (Kalantar‐Zadeh et al. 2010).

Based on the evidence that luminal phosphate concentrations in the small intestine are in the low millimolar range, our data suggest that transepithelial phosphate absorption in vivo is predominately Na^+^‐independent, with Na^+^‐dependent (presumably NaPi‐IIb‐mediated) transport playing a lesser role than currently proposed. For example, while the present study confirms that phosphate absorption in the rat occurs maximally in the jejunum (Walling 1977; Marks et al. 2006; Giral et al. 2009), we also demonstrate that only 30% of total transport in vivo can be resolved into a Na^+^‐dependent component. This is in keeping with studies using NaPi‐IIb^−/−^ knockout mice, which showed that under in vivo conditions the acute postprandial change in serum phosphate was reduced by only 45–50% relative to that observed in wild‐type animals (Sabbagh et al. 2009). The variation in the overall contribution of Na^+^‐dependent phosphate transport in these two studies may be due to the different species used, but is more likely to reflect the difficulty in accurately estimating this component of transport using Na^+^‐free buffers. This may also explain why there seemed to be a lower proportion of Na^+^‐dependent transport in the jejunum in vivo compared with in vitro, since secretion of Na^+^ was detectable in luminal Na^+^‐free buffers taken at the end of the in vivo*,* but not the in vitro experiments. It might also explain the findings of Williams and DeLuca (2007) who suggested that intestinal phosphate absorption showed no Na^+^‐dependency in vivo after measuring ^33^P appearance in serum 60 min after gastric gavage with NaH_2_PO_4_ or KH_2_PO_4_ (Williams and DeLuca 2007). However, secretion of Na^+^ into the luminal fluid in vivo is unlikely to explain the apparent complete absence of Na^+^‐dependent phosphate transport in the duodenum compared with the ~50% seen in vitro.

An alternative explanation for the disparity in the overall contribution of Na^+^‐dependent and ‐independent transport observed in vivo versus in vitro may reflect the differences in “measurable” paracellular transport using these very different experimental conditions. We suggest that the everted sleeve technique is a valid and useful measure of phosphate transport across the BBM of intact epithelium, since Na^+^‐dependent transport is readily detectable, particularly at phosphate concentrations in keeping with the affinity of NaPi‐IIb for phosphate. However, examining phosphate absorption in vivo may be more physiologically relevant and reveal regulatory changes in the dominant Na^+^‐independent transport pathway. In this context, while tight junction proteins are known to provide cell adhesion between epithelial cells, some claudin isoforms can also function as channels that are regulated by signal transduction pathways to provide a selective paracellular route for passive ion flow (Amasheh et al. 2011). Whether claudins can selectively influence paracellular phosphate transport has not been investigated, but they do appear to play a role in the absorption of calcium in the small intestine (Fujita et al. 2008). It is possible that the unique expression profiles of the different claudin isoforms along the intestinal tract (Fujita et al. 2006; Markov et al. 2010), and the resulting differences in paracellular permeability, could explain the differences in Na^+^‐independent transport in different intestinal segments in vivo.

Another alternative explanation is that an as yet unidentified pathway for transcellular Na^+^‐independent phosphate transport may exist, particularly in light of the relatively high levels of Na^+^‐independent transport seen at millimolar phosphate concentrations in our in vitro studies. In this context, lessons may be learnt from our knowledge of phosphate handling in ruminants, where, in contrast to monogastric animals, the gastrointestinal tract plays a more important role in the maintenance of phosphate balance than renal excretion. Levels of phosphate found in the ruminant gastrointestinal tract are in the range of 16–40 mmol/L, which originates mainly from salivary secretions, rather than dietary intake (Shirazi‐Beechey et al. 1991). Interestingly, there have been reported differences in the profile of phosphate absorption in different ruminant species: in sheep, the highest rate of phosphate absorption is found in the ileum, with lower levels occurring in the proximal segments of the small intestine and in the proximal colon; in contrast, phosphate absorption is confined to the duodenum and jejunum in goats (Schroder et al. 1995). Sodium‐dependent phosphate transport in goat jejunum is mirrored by the presence of NaPi‐IIb mRNA and protein, and is upregulated by dietary phosphate restriction (Huber et al. 2002). While in the duodenum of goats, NaPi‐IIb is absent (Huber et al. 2002) and phosphate transport is mediated by an H^+^‐dependent transport process (Shirazi‐Beechey et al. 1996) that has a 10‐fold higher *K*_m_ than jejunal NaPi‐IIb‐mediated transport and is not affected by dietary phosphate restriction (Huber et al. 2002). It is also worth noting that recent studies using the human intestinal cell line, Caco2BBE, have shown that phosphate transport in this cell type is solely Na^+^‐independent. Transport can be resolved into more than one Na^+^‐independent transport system depending on the level of phosphate present in the growth medium, at least one of which is an H^+^‐dependent process (Candeal et al. 2014). In addition, incubation of these cells with high levels of phosphate promotes Na^+^‐independent phosphate uptake which is dependent on de novo RNA and protein synthesis (Candeal et al. 2014).

The importance of the ileum in intestinal phosphate uptake is unclear: while some studies have reported that the rat ileum has little or no capacity to absorb phosphate (Peters and Binswanger 1988; Marks et al. 2006), others have reported that the magnitude of phosphate absorption is similar in the duodenum, jejunum and ileum, perhaps in vivo attributable in part to differences in length (surface area for absorption) and transit time (resident time for absorption) (Cramer 1961; Kayne et al. 1993). Our findings demonstrate minimal ileal phosphate transport using 0.1 mmol/L luminal phosphate, but at physiologically relevant levels of phosphate there is significant transport in vivo*,* with total transepithelial absorption occurring at rates similar to those seen in the duodenum. As already suggested, because the transit time in rodents is ~120 min in the ileum versus ~2 min in the duodenum (Duflos et al. 1995), it is likely that this segment contributes significantly to phosphate absorption (Cramer 1961; Kayne et al. 1993). However, surprisingly significant Na^+^‐dependent phosphate transport was demonstrated in the ileum in vivo*,* but not in vitro, when using millimolar phosphate concentrations. If the everted sleeve method is accepted to reflect transport processes at the BBM, these findings correlate with the absence of NaPi‐IIb and PiT1 protein expression at the ileal BBM in the rat (Marks et al. 2006; Giral et al. 2009), and suggest that an additional, and as yet uncharacterized, Na^+^‐dependent transporter may be present in this region, which is only revealed when measuring transepithelial phosphate absorption in vivo.

We have also provided evidence that the distal, but not proximal, colon can absorb phosphate. The finding that Na^+^‐dependent transport is detectable in vitro corresponds with the presence of NaPi‐IIb gene transcript in the colon (Hilfiker et al. 1998). However, this pathway is unlikely to be significant in phosphate absorption in this segment, because in vivo Na^+^‐independent transport predominates. The importance of these findings to overall phosphate balance is uncertain, since increasing solidity of distal colonic contents would make soluble phosphate less accessible for absorption. However, our finding of significant Na^+^‐independent uptake at high luminal phosphate concentrations in the distal colon is probably relevant to the clinical observation, and complication, of hyperphosphatemia following the use of high phosphate‐containing enemas (Hunter et al. 1993; Hu et al. 1997; Carl and Mitchell 2007).

In summary, we have shown that phosphate concentrations in the intestinal lumen are typically in the low millimolar range and that under these conditions Na^+^‐independent transport is likely to be the predominant pathway for phosphate absorption in vivo. Previous studies of the regulation of intestinal phosphate transport have used in the main in vitro techniques to assess changes in Na^+^‐dependent phosphate transport, which has been attributed to NaPi‐IIb; yet the contribution of Na^+^‐dependent transport to overall phosphate transport in vivo seems to be relatively small. Our study highlights the value and importance of using both approaches to study phosphate transport. Indeed, the use of a single method may explain the discrepancies in the literature concerning the mechanisms involved in phosphate transport. However, additional studies are required to gain more insight into the mechanism(s) and regulation of Na^+^‐independent phosphate absorption in the jejunum and colon, as well as the proteins responsible for Na^+^‐dependent uptake at phosphate levels normally present in the ileum.

## Conflict of Interest

We thank Acologix Inc, particularly Dr David Rosen, for support of this project. RJU is currently on secondment as a Chief Scientist to AstraZeneca CVMD iMed R&D, Mölndal, Sweden. No conflicts of interests are declared by the author(s).
